# Antidepressant and antipsychotic use in an Italian pediatric population

**DOI:** 10.1186/1471-2431-11-40

**Published:** 2011-05-23

**Authors:** Antonio Clavenna, Margherita Andretta, Paola Pilati, Maurizio Dusi, Michele Gangemi, Maria Beatrice Gattoni, Giuseppe Lombardo, Leonardo Zoccante, Luigi Mezzalira, Maurizio Bonati

**Affiliations:** 1 Laboratory for Mother and Child Health, Department of Public Health, Mario Negri Institute for Pharmacological Research, Milan, Italy; 2 UOC Servizio Farmaceutico, Azienda ULSS 20, Verona, Italy; 3 Ospedale Villa Santa Giuliana, Verona, Italy; 4 Azienda ULSS 20, Verona, Italy; 5 U.O. Neuropsichiatria Infantile, Policlinico G.B. Rossi, Verona, Italy

## Abstract

**Background:**

The safety and effectiveness of psychotropic drug use in the paediatric population is widely debated, in particular because of the lack of data concerning long term effects.

In Italy the prevalence of psychotropic drug prescriptions increased in the early 2000s and decreased afterwards. In such a context, a study with the aim to estimate the incidence and prevalence of psychotropic drug prescription in the paediatric population and to describe diagnostic and therapeutic approaches was performed.

**Methods:**

The study population was composed of 76,000 youths less than 18 years and living in the area covered by the local health unit of Verona, Italy. The data source was the Verona local health unit's administrative prescription database. Prevalence and incidence of antidepressant and/or antipsychotic drug prescriptions in the 2004-2008 period were estimated. Children and adolescents receiving antidepressant and/or antipsychotic drug prescriptions between 1 January 2005 and 31 December 2006 were identified and questionnaires were sent to the prescribers with the aim to collect data concerning diagnostic and therapeutic approaches, and care strategies.

**Results:**

The prevalence of psychotropic drug prescriptions did not change in the 2004-2008 period, while incidence slightly increased (from 7.0 in 2005 to 8.3 per 10,000 in 2008). Between 1 January 2005 and 31 December 2006, 111 youths received at least one psychotropic drug prescription, 91 of whom received antidepressants. Only 28 patients attended child and adolescent psychiatry services. Information concerning diagnostic and therapeutic approaches, and care strategies was collected for 52 patients (47%). Anxiety-depressive syndrome and attention disorders were the diseases for which psychotropic drugs were most commonly prescribed. In all, 75% youths also received psychological support and 20% were prescribed drugs for 2 or more years.

**Conclusions:**

Despite the low drug prescription prevalence, the finding that most children were not cared for by child and adolescent psychiatric services is of concern and calls for a systematic, continuous monitoring of psychopharmacological treatments.

## Background

Safety and efficacy of psychotropic drug use in the paediatric population have been widely debated, in particular due to the lack of data concerning long term effects [1,2]. Moreover, a link between the use of selective serotonin reuptake inhibitors (SSRIs) and increased risk of suicide ideation has been documented in the paediatric population and in young adults[3,4].

Considering antidepressants, the benefit of SSRIs seems to be greater in general anxiety disorders, intermediate in obsessive compulsive disorder (OCD), and modest in major depressive disorder (MDD) [5].

Moreover, the efficacy of pharmacological therapies for MDD in children and adolescents is controversial [1]. A systematic review of the literature did not find a statistically significant difference between tricyclic antidepressants and placebo [6]. In addition, taking SSRIs into account, the risk-benefit profile appears favourable only for fluoxetine [5,7]. Children with MDD were found to be more responsive to placebo compared to children with other neuropsychiatric disorders and this finding may, at least partly, explain the low efficacy of antidepressants [8].

Randomised controlled trials (RCTs) evaluating the efficacy of antipsychotics mainly involved the second generation drugs. In particular, a systematic review concerning the treatment of psychosis and bipolar disorder in children and adolescents found a total of 18 RCTs that evaluated the efficacy of olanzapine (7 trials), risperidone and quetiapine (5), clozapine (4), and aripiprazole (3) [9].

The only two first generation antipsychotics for which RCTs are available are haloperidol (mainly for schizophrenia and Tourette's syndrome) and pimozide (Tourette's syndrome). The evidence on the efficacy of the other first generation antipsychotics is scant and limited to case series or uncontrolled studies [10].

A Cochrane Collaboration systematic review compared risperidone to placebo in children with autism spectrum disorder, documenting its efficacy in decreasing behavioral symptoms (irritability and aggressive behavior) [11]. Risperidone was found to be effective also in reducing symptoms in children and adolescents with disruptive behavior. Fewer data are available concerning the efficacy of other antipsychotics in pervasive and disruptive behavior [12].

However, in evaluating the safety and efficacy of psychotropic drugs, the chance that results may be biased by conflicts of interests should be taken into account [13].

Few psychotropic drugs are licensed for use in children in Italy, and in Europe in general, and for some diseases only. Sertraline and fluvoxamine are licensed in Italy and in most of the European countries for the treatment of OCD (in children ≥ 6 years), fluoxetine is licensed for MDD in children ≥ 8 years, and imipramine is licensed in Italy for children ≥ 6 years, but for nocturnal enuresis only.

Haloperidol, periciazine, risperidone, chlorpromazine, and levomepromazine are the antipsychotics licensed for use in children in Italy. However, the Summary of Product Characteristics reports the paediatric dosage schedule only for the first three drugs. Aripiprazole was licensed in 2009 by the European Medicines Agency for the treatment of schizophrenia in adolescents over 15 years old. In the United States, but not in Europe, the second generation antipsychotics olanzapine and quetiapine are also licensed for use in children.

The prevalence of psychotropic drug prescriptions in the paediatric population differs among countries, with the highest values in the United States [14-17]. In a three-country comparison study, the psychotropic medication prevalence in 2000 was 6.7% in the United States, 2.9% in the Netherlands, and 2.0% in Germany [15]. A psychotropic drug prescription prevalence of 2.2% was estimated in France in 2004 [16], and one of 4.9% in Iceland in 2007 [17].

In Italy, the drug prescription prevalence increased in the 2000-2002 period, in particular the prevalence of SSRIs, and decreased afterwards, reaching a value of around 2 per 1,000 youths under 18 years in 2004, with differences between geographical settings [18]. In fact, in a sample of 27 Italian local health units the prevalence ranged from 0.8‰ to 6‰ [18].

Drug utilization studies performed in Italy analysed data collected in prescription databases, with some limitations, mainly: the lack of information concerning the diseases for which drugs were prescribed, the use of non reimbursed drugs, and the access to non pharmacological therapies.

In this context, a retrospective study was performed in a sample of children and adolescents treated with psychotropic drugs, in an homogeneous geographical context (the local health unit, LHU, of Verona, in northern Italy), with the aim to evaluate the diagnostic and therapeutic approaches in the management of psychiatric disorders.

## Methods

Psychotropic drug prescriptions dispensed in the 2004-2008 period to children and adolescents < 18 years old living in the area covered by the Verona local health unit (LHU) were analysed. The data source was the Verona LHU's administrative prescription database that stores all community (i.e. outside hospital) prescriptions reimbursed by the National Health Service (NHS). The structure of the database has been described in detail elsewhere [19]

Psychotropic drugs were classified according to the World Health Organization categories and comprised the following subgroups of the Anatomic Therapeutic Chemical (ATC) classification system: antidepressants (N06A) and antipsychotics (N05A). Anticonvulsants were excluded since in children they are mainly used to treat epilepsy, while anxiolytics were excluded because they are not reimbursed by the Italian National Health Service and were thus not present in the prescription database. Stimulants were not taken into account since methylphenidate and atomoxetine were marketed in Italy only in 2007.

The prescription trend in the 2004-2008 period was analyzed, and the annual prevalence and incidence were calculated. Prevalence was defined as the number of individuals who received at least one psychotropic drug prescription per 10,000 individuals in the population, and incidence was defined as the number of people who received a psychotropic drug prescription for the first time per 10,000 youths in the population. Incident users were defined as youths who did not receive any psychotropic drug prescription in the preceding consecutive 12 months.

Using the prescription database, children and adolescents who received a psychotropic drug in the 2005-2006 period were identified and, in November 2007, a previously validated questionnaire was sent to the primary care physician (family paediatrician or general practitioner) who cared for the youth.

Youths were selected using the national identification number (the so called fiscal code, an alphanumeric univocal code). The selection of the sample was performed by a pharmacist in Verona LHU, who usually accessed the prescription database for administrative reasons. Each physician received a list reporting the fiscal code numbers of the children and adolescent in his/her charge, with the aim to allow him/her to identify the patient and fill in the questionnaire. Only the physician who cared for the child was able to identify the patient. Data concerning diagnostic approaches, pharmacological and non pharmacological therapies, neuropsychiatric disorders for which drugs were prescribed, hospitalizations, and side effects were collected. Moreover, the outcome was evaluated using the clinical global impression improvement (CGI-I) scale. A similar questionnaire, regarding the same previously identified youths, was also sent to child psychiatrists working in Verona outpatient and hospital child and adolescent neuropsychiatry services.

In Italy, children are assigned to a family paediatrician until they are 6 years old; afterwards, the parents can choose to remain with that paediatrician until child is 14 years old or to register the child with a general practitioner. All adolescents over 14 years of age are assigned to a general practitioner.

Child and adolescent neuropsychiatry services exist at the hospital and community level to care for children and adolescents with neurologic and/or psychiatric disorders and for their families [20].

The study was approved by the Verona LHU's Ethics Review Board. Data were analysed using an anonymous patient code, study specific and different from the fiscal code.

A χ^2 ^test was performed in order to compare the profile of children receiving recurrent (>1 prescriptions) with children receiving occasional prescriptions.

Significance of the prevalence linear trend (χ^2 ^trend) was assessed across years.

A P value < 0.05 was considered statistically significant.

## Results

### Prevalence and incidence of psychotropic drug prescriptions

The prevalence of psychotropic drug prescriptions did not change in the 2004-2008 period (χ^2 ^t = 0.08; p = 0.78). The incidence slightly increased, from 7.0 per 10,000 in 2005 to 8.3 per 10,000 in 2008, but the increase was not statistically significant (χ^2 ^t = 1.32; p = 0.25).

**Figure 1:**
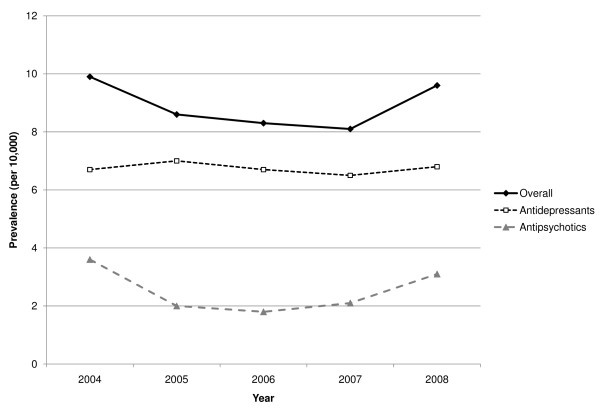
Trend of psychotropic drug prescription prevalence in the 2004-2008 period in the Verona local health unit.

While the prevalence of antidepressants was quite stable, the prevalence of antipsychotics decreased in the 2004-2006 period and then increased to a value similar to that of 2004. (Figure 1)

Citalopram (1.3 per 10,000 youths), sertraline (1.2 per 10,000 youths), and paroxetine (1.1 per 10,000) were the most prescribed psychotropic drugs in 2004, while paroxetine (1.5 per 10,000), sertraline (1.5 per 10,000), and escitalopram (1.0 per 10,000) were the drugs most commonly prescribed in 2008.

In the 2005-2006 period 111 children and adolescents (54 boys and 57 girls) 2-17 years old received at least one psychotropic drug prescription: 86 of them received antidepressants, 20 antipsychotics, and 5 both. A total of 14 youths received prescriptions in either 2005 or 2006.

**Figure 2:**
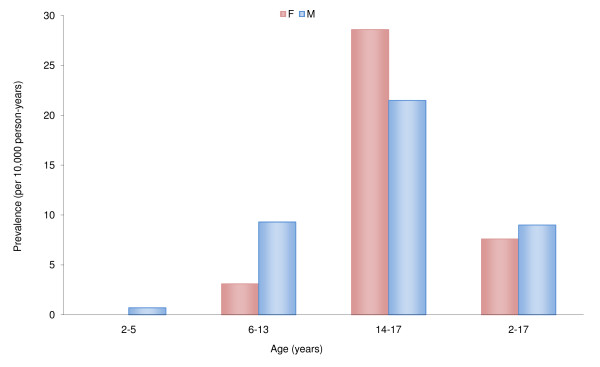
Prevalence of psychotropic drug prescriptions (per 10,000 person-years) by gender and age.

Figure 2 reports the distribution of drug prescription prevalence by gender and age. Prevalence increased with increasing age, with the highest value in adolescent girls (28 per 10,000 person-years; 95%CI 27-29 per 10,000 person-year).

A total of 25 different drugs were prescribed. Those most commonly prescribed were sertraline and amitryptiline (14% of the youths), paroxetine (12%), and citalopram (10%). Haloperidol was the most commonly prescribed antipsychotic (8%).

Every child received a mean of 2.4 prescriptions (median: 1; interquartile range, IQR: 1-3) and 3.7 packages (median: 2; IQR: 1-5).

In all, 56 youths received only 1 prescription (occasional prescription) and 39 youths received one medication package only during the 2-year observation period. No differences were found between youths receiving single versus recurrent prescriptions for age, gender, or drug class.

In all, 62% of children and adolescents received a drug not licensed for use in children.

Ninety physicians cared for 111 youths. More specifically, 69 general practitioners cared for 89 patients, while 21 family paediatricians cared for 22 children. General practitioners and paediatricians prescribed psychotropic drugs to 90 patients (81%), while for 21 youths (19%) drugs were prescribed by a child psychiatrist or psychiatrist. In all, 28 children attended a child psychiatry service; the percentage of children attending such a service was higher among youths with recurrent prescriptions (33% versus 18%).

## Results of the survey

### Profile of responders

Data from questionnaires were available for 52 patients, 31 of which received recurrent prescriptions.

Youths for whom questionnaires were available were younger than youths for whom information was not provided: the age expressed as mean ± standard deviation was 13.5 ± 3.1 years versus 15.2 ± 3.3 years, respectively (p = 0.02). In particular, it was possible to collect data only for 37% of the adolescents compared with 73% of the younger children (χ^2 ^= 10.3, p = 0.0006). No statistically significant differences were found between gender.

Children included in the survey received a greater number of prescriptions during the 2 year observation period: the mean number of prescriptions (± standard deviation) was 3.6 ± 5.7 versus 1.9 ± 1.4 in children for whom data were not provided (p = 0.002).

The percentage of children with recurrent prescriptions included in the survey was slightly higher than that of children with one prescription only (56 versus 38%, p = 0.05). For 26 patients the questionnaire was filled in by the primary care physician and for 26 by the child psychiatrist (for 20 by both types of physicians).

### Therapy

A total of 38 patients received antidepressants (43% of antidepressant users) and 17 antipsychotics (65%), while 3 received both.

23 children and adolescents received psychotropic drug prescriptions also during 2007: 14 were treated with antidepressants, and 11 with antipsychotics, 2 with both. In all, 39 patients (75%) received a psychological therapy in the 2005-2006 period, 18 of whom received it also during 2007.

Seven youths reported side effects to their physician (5 in therapy with antipsychotics and 2 with antidepressants), in particular: increased appetite (3), drowsiness, dyskinesia, irritability, and seizures (1 each). Only 1 patient stopped treatment because of side effects.

### Diagnostic and care pathways

Anxiety-depressive disorders (33% of the cases) and attention deficit hyperactivity disorder (ADHD) (21%) were the disorders for which drugs were more commonly prescribed. The prevalence of anxiety-depressive disorders treated with drugs in Verona's LHU was estimated at 2.4 cases per 10,000 (95%CI 2.3-2.5 per 10,000) and the prevalence of ADHD at 1.4 per 10,000 (95%CI 1.3-1.5 per 10,000).

The diagnosis was made for the first time by a child psychiatrist in 73% of the cases. Sixteen patients were visited by at least one other specialist before the diagnosis was made and 18 were visited by other specialists after the diagnosis was made. In all, 10 youths were visited by 3 or more specialists.

During 2007, 21% of the patients were followed by paediatricians or general practitioners only.

### Outcome

According to the physician's judgment, the symptoms of 73% of the youths resolved partially or completely.

For 24 out of 52 patients (46%) the disorder was rated by the physician as improved/very improved (CGI-I 1 or 2), for 19 there was no change or minimal improvement, while only in 3 cases the disease worsened.

In all, 18 youths considered as improved/very improved were taking antidepressants (out of 38 treated with these medications; 47.4%) and 6 were taking antipsychotics (out of 17; 35.3%). Thirteen of 24 children (54%) received recurrent prescriptions in the 2005-2006 period.

Two patients treated with antidepressants (citalopram, fluvoxamine) and 2 treated with antipsychotics (olanzapine, clotiapine) attempted suicide. All 4 patients were adolescents: 3 male and 1 female.

Thirty-one patients (60%) experienced some difficulties in their school performances: 9 had ADHD and 9 anxiety-depressive disorder.

### Psychiatric disorders in the family members

In 15 cases, at least one parent had a psychiatric disorder and in 12 cases at least one family member took psychotropic drugs (antidepressants and/or benzodiazepines). The chance to have a family member with a psychiatric disorder was greater in youths with recurrent prescriptions compared with youths with occasional prescriptions (42 versus 10%; chi square 4.9; p = 0.03).

## Discussion

In Verona LHU's large population, the prevalence of psychotropic drug prescriptions was lower than in Italy as a whole [18,21] and in other countries [14-17].

Drug utilization studies found a greater prescription prevalence of antidepressants in the United States (10%), followed by Iceland (2.3%), while in other European countries the antidepressant prescription prevalence ranged from 0.2 to 0.6%[14-17,22,23]. A similar pattern was observed for antipsychotics, with a prevalence ranging from 0.7 per 1,000 in Italy to more than 1% in the United States and Iceland [14-17,24].

The prevalence by gender and age is consistent with the findings of other drug utilization studies and with the epidemiological features of the disorders.

Some data need to be commented on. First of all, the fact that antidepressants are prescribed occasionally: 37% of youths received one medication package only during the 2-year observation period, which was not sufficient to cover an 8 week treatment period.

Paroxetine, citalopram, and escitalopram are among the most prescribed antidepressants, despite the fact that they are not licensed for use in children. Moreover, the evidence on efficacy is scant and warnings were issued by Italian and international drug regulatory agencies concerning the risk of increased risk of suicide ideation [25,26]. In particular, it is interesting to note that the prevalence of paroxetine increased slightly over time, while the prevalence of escitalopram doubled from 2005 to 2008, and this drug became the third most prescribed psychotropic drug in 2008. This finding is consistent with the use of antidepressants in the adult population, where escitalopram was the most prescribed drug [27], but only 2 randomised controlled trials evaluated the safety and efficacy of this drug in the paediatric population, with conflicting results [28,29].

In contrast, fluoxetine, the only SSRI licensed for MDD and for which evidence of efficacy exists [5-7], was scantly prescribed.

Unfortunately, although a few reminders were sent to physicians, there was a low response rate to the questionnaire; in particular there was a lower compliance by general practitioners, and concerning adolescents.

However, children included in the survey received a greater number of prescriptions during the 2 year observation period and, therefore, it may be hypothesized that most of the patients for which data were not provided had mild acute disorder (e.g. anxiety) managed exclusively by the general practitioners, while the survey was able to capture patients with chronic disorders. The greater percentage of youths with recurrent prescription included in the survey (physicians' responders) may support this hypothesis. Moreover, it was possible to collect data for all 14 youths who received drug prescriptions in either 2005 or 2006.

In all, 47% of the youths resulted still in therapy when data collection began; considering youths who were receiving psychotropic drug prescriptions during 2005, the rate was 20%.

In nearly all cases the drug therapy was integrated with a psychological treatment, as recommended by international treatment guidelines [30].

On the contrary, only 28 children and adolescents (25% of psychotropic users) attended child psychiatric services. This fact underlines a low adherence to the standard care approach.

In ¾ of the cases a partial or complete resolution of symptoms was reported by the physicians, even though only for 46% of the patients the CGI-I was < 3 (very much improved or improved). The percentage decreased to 35% when considering the youths with recurrent prescriptions.

Almost all patients were visited by a child psychiatrist, nearly half of them by 2, and 20% by at least three different specialists. The frequent request for a second (or third) opinion may reflect the disease complexity and the parents' difficulties in facing the disorder.

The main strength of this study was the possibility to collect data concerning the disease for which drugs were prescribed.

As expected, anxiety-depressive disorders and attention deficit disorders were the diseases for which drugs were more commonly prescribed, and covered half of the children receiving psychotropic drug prescriptions.

The prevalence of pharmacologically treated anxiety-depressive disorder was roughly estimated at 2.4 per 10,000 and the prevalence of ADHD at 1.4 per 10,000. For both diseases these estimates are lower than expected on the basis of the epidemiological data collected at national and international levels [14,31], and underline the need for prospective studies with the aim to better define the burden and the prevalence of these diseases. The prevalence of drug treated patients may be underestimated, in particular for ADHD since methylphenidate and atomoxetine were not licensed in Italy before 2007. The estimate in this study is, however, consistent with an analysis of data collected in the national registry that monitors children treated with methylphenidate or atomoxetine (set up in 2007, when the drugs became available on the market), with an estimate of 1.5 cases per 10,000 in the Lombardy region [32]. However, the rough estimate of children requiring pharmacological treatment for ADHD in Italy is 66 per 10,000 [32], and it is therefore possible that many children with ADHD in Verona did not receive the appropriate therapy for the disease.

Despite the small sample and the fact that the study was not aimed to investigate drug safety, it should be noted that 7 out of 52 youths experienced side effects, all of which were known and previously reported [33,34]. The fact that 4 cases of suicide attempt or ideation were reported should also be highlighted, even if it is not possible to discriminate between the role of the disease and that of the drugs.

One third of the youths had at least one family member with a psychiatric disorder and/or in therapy with psychotropic drugs. This study did not permit an analysis of whether this percentage is higher than in families without psychiatric disorders. However, some differences were observed between patients with recurrent versus occasional prescriptions, indicating that the prevalence of severe psychiatric disorders may be higher among youths whose parents have psychiatric disorders [35,36].

### Strengths and limitations

This is the first Italian study investigating the diagnostic and therapeutic approaches in children and adolescents treated with psychotropic drugs.

There are some limitations, however. First of all, this study was performed in a local setting (even if quite large) and it may not described the national situation, both in terms of prescription prevalence and approaches. Moreover, the number of youths with a psychotropic drug prescription was small, and only for half of them it was possible to obtain data concerning the diagnostic and therapeutic approaches due to the low physician response rate. It is likely that many data concerning prescriptions made by primary care physicians without the child psychiatrist's advice (e.g. SSRIs for anxiety syndrome in adolescents) were missed. Finally, it was not possible to collect systematically information concerning the treatment duration or dosage, the number of medical visits, or the kind of psychotherapy performed.

## Conclusions

Despite some limitations, this study highlights that there is a need to define (and to comply with) appropriate diagnostic and therapeutic approaches for child and adolescent psychiatric disorders. In most instances, psychotropic drugs were prescribed by general practitioners without the advice of child psychiatrists, as occasional treatment of acute disorders, using drugs not licensed for use in the paediatric population.

Educational interventions with the aim to improve the rational use of psychotropic drugs in children and adolescents are therefore needed.

## Competing interests

The authors declare that they have no competing interests

## Authors' contributions

All the authors contributed equally to the design of the study and in writing the protocol. MA and PP collected the data. AC undertook the statistical analysis and wrote the first draft of the manuscript. LM and MB supervised the study. All authors contributed to and have approved the final manuscript.

## Pre-publication history

The pre-publication history for this paper can be accessed here:

http://www.biomedcentral.com/1471-2431/11/40/prepub
